# Resource Needs for the Trivalent Oral Polio to Bivalent Oral Polio Vaccine Switch in Indonesia

**DOI:** 10.1093/infdis/jix073

**Published:** 2017-06-30

**Authors:** Marionette Holmes, Taiwo Abimbola, Merry Lusiana, Sarah Pallas, Lee M. Hampton, Retno Widyastuti, Idawati Muas, Karlina Karlina, Soewarta Kosen

**Affiliations:** 1 Global Immunization Division, US Centers for Disease Control and Prevention, and; 2 Economics Department, Spelman College, Atlanta, Georgia; and; 3 Policy Unit, Ministry of Health, Jakarta, Indonesia

**Keywords:** vaccine switch, cost analysis, resource utilization, Indonesia.

## Abstract

**Background.:**

We present an empirical economic cost analysis of the April 2016 switch from trivalent (tOPV) to bivalent (bOPV) oral polio vaccine at the national-level and 3 provinces (Bali, West Sumatera and Nusa Tenggara) for Indonesia’s Expanded Program on Immunization.

**Methods.:**

Data on the quantity and prices of resources used in the 4 World Health Organization guideline phases of the switch were collected at the national-level and in each of the sampled provinces, cities/districts, and health facilities. Costs were calculated as the sum of the value of resources reportedly used in each sampled unit by switch phase.

**Results.:**

Estimated national-level costs were $46 791. Costs by health system level varied from $9062 to $34 256 at the province-level, from $4576 to $11 936 at the district-level , and from $3488 to $29 175 at the city-level. Estimated national costs ranged from $4 076 446 (Bali, minimum cost scenario) to $28 120 700 (West Sumatera, maximum cost scenario).

**Conclusions.:**

Our findings suggest that the majority of tPOV to bOPV switch costs were borne at the subnational level. Considerable variation in reported costs among health system levels surveyed indicates a need for flexibility in budgeting for globally synchronized public health activities.

Following the declaration of the global eradication of type 2 wild polio virus (WPV2) in 2015, the continued use of trivalent oral polio vaccine (tOPV), which contains attenuated types 1, 2, and 3 polioviruses, posed an unacceptable risk for creating new type 2 circulating vaccine-derived polio viruses (cVDPV2s) that could cause paralytic poliomyelitis [1–3]. To address this risk, the World Health Organization (WHO) Strategic Advisory Group of Experts on Immunization recommended that all countries using tOPV replace the vaccine with bivalent oral polio vaccine (bOPV), which only contains attenuated types 1 and 3 polioviruses, during April 2016 [4]. This globally synchronized switch from tOPV to bOPV required coordinated planning and action at global, regional, national, and subnational levels, with the potential for significant additional costs to countries for planning new policies, health-staff training, supply-chain adjustment, vaccine disposal/recall, and validation of the switch.

The gradual replacement of an existing vaccine with a new vaccine should normally have minimal cost implications because the existing vaccine would just be used up and then replaced as the new vaccine was supplied in its place. The globally synchronized switch from tOPV to bOPV was unusual because it required that all tOPV use stop during a window of a few weeks detailed in the WHO global switch implementation guidelines, that all remaining tOPV be recalled and promptly destroyed, and that bOPV be distributed and its use start during the same window of a few weeks [5]. The Global Polio Eradiation Initiative (GPEI) provided $23.7 million in assistance to pay for the switch in select countries at highest risk for a cVDPV2 outbreak or with the greatest financial need [6]. The WHO switch guidelines encouraged countries to establish a new “national switch management committee [that] is responsible for securing funds” that could be used to hire additional staff and cover additional costs associated with the switch ([7], section 3.3, page 16).

National immunization programs developed estimated budgets during the process of planning the switch with the help of coordinated multistakeholder regional and global technical assistance. These budgets informed the allocation of funding from the GPEI to countries to support the costs of the switch. Although the normative prospective budget projections provided an important basis for resource allocation, empirical data on the expenditures and value of in-kind resources used in implementing the switch provide an estimate of the actual costs of the switch. Such retrospective empirical cost estimates can provide valuable information for planning the resources required for potential future vaccine switches or withdrawals (eg, a switch from bOPV to monovalent oral polio vaccine 1 or the future withdrawal of bOPV).

Accordingly, this article presents results from a retrospective empirical economic cost analysis of tOPV–bOPV switch planning, implementation, and validation in Indonesia. Indonesia was chosen for this case study for several reasons. Its large population, complex geographic landscape, and middle-income status with a per capita income of $3346 made it an important test case for the switch [8]; resource requirements in such an environment are expected to represent a potential upper bound on switch costs compared with lower-income countries or countries with more compact geographies and smaller population sizes. In addition, although Indonesia’s last identified polio case caused by indigenous wild poliovirus (WPV) was in East Java in 1995, the ongoing risk of poliovirus importations is indicated by 351 polio cases caused by imported type 1 WPV (WPV1) during 2005–2006 and 46 polio cases caused by indigenous type 1 circulating vaccine-derived poliovirus (cVDPV1) detected in 2005 [9–11]. Indonesia has actively implemented the Polio Eradication and Endgame Strategic Plan since 2013 [12]. In 2015, the Ministry of Health developed the Indonesia National Switch Plan (INSP), adapted from WHO’s global switch implementation guidelines [13]. The plan involved all health facilities ceasing use of tOPV and beginning use of bOPV in April 2016, with Biofarma, a parastatal vaccine manufacturer owned by the Government of Indonesia, both supplying the new bOPV and destroying the old tOPV.

This article addresses the following question: What were empirical economic costs of the tOPV to bOPV switch in Indonesia? We present costs by (1) health system level (national, provincial, district/city, and health facility [HF]); (2) switch phase as identified in the WHO global switch guidelines; and (3) estimated aggregate costs by sampled province. As a case study of a single country, our findings highlight the operational features of implementing a global initiative at national and subnational levels and suggest directions for estimating resource needs and allocating resources to support similar vaccine switches and withdrawals in the future.

The evaluation protocol was determined to be not human subjects research and therefore exempt from institutional review board (IRB) review by the CDC Center for Global Health. The evaluation protocol was reviewed by the IRB of the National Institute of Health Research and Development, Indonesian Ministry of Health and determined to be exempt from further IRB review (no. LB.02.01/5.2/KE.267/2016).

## METHODS

### Site Sample

We estimated the incremental empirical economic cost of the switch from tOPV to bOPV at the national level and in 3 selected provinces for Indonesia’s Expanded Program on Immunization (EPI). We used the methodology as put forth by EPI Costing (EPIC) Common Approach to stratify the provinces [14, 15]. A convenience sample of 3 provinces was selected: Bali (95.9% oral polio type 3 [OPV3] coverage), West Sumatra (83.8% OPV3 coverage), and Nusa Tenggara Timur (NTT) (<75% OPV3 coverage) (Table 1). The 3 selected provinces (Table 1) were not intended to be representative of all 34 provinces in Indonesia.

**Table 1.** Oral Polio Vaccine Coverage in Sampled Provinces, Cities, and Districts^1^

Geographical area	No. of infants (aged <1 y)	4th Series (OPV3) coverage rate
Province (high coverage): Bali	70392	95.9%
City^2^: Kota Denpasar	18763	96.1%
District^3^: Karangasem	8550	91.1%
Province (medium coverage): Sumatera Barat (West Sumatra)	109336	83.8%
City: Kota Padang	3615	95.0%
District: Padang Pariaman	18845	84.8%
Province (low coverage): Nusa Tenggara Barat	125752	67.6%
City: Kota Kupang	11162	79.4%
District: Timor Tengah Selatan	10861	88.2%

Statistics are based on Expanded Progam on Immunization national program office 2015 immunization coverage. High provincial coverage = OPV3 immunization coverage among infants >90%. Medium provincial coverage = OPV3 immunization coverage among infants of 75%–90%. Low provincial coverage = OPV3 immunization coverage among infants <75%. City = urban area^.^ District = rural area.

Abbreviation: OPV3, oral polio type 3.

Within selected provinces, we stratified according to urban area (cities) and rural area (districts) and selected a convenience sample of 1 district and 1 city within each province. Districts are the next lowest level of health-system administration below the provincial level. Finally, within each city and district, we stratified (where possible) according to HF ownership (either publicly owned by the Indonesian government or privately owned for-profit or not-for-profit) and selected a convenience sample of 1 public and 1 private HF. Private HFs within the sampled rural settings were rare; in the absence of a private HF, we sampled 2 government HFs. In each province, we sampled 4 HFs, including 1 public and 1 private HF for each city; all rural district HFs were public except 1 private HF in Bali province.

### Switch Phases

We collected costs for the 4 major phases of the switch defined by the WHO global switch guidelines—(1) plan, (2) prepare, (3) implement, and (4) validate—using a standard activity-based costing method [5, 16, 17]. In Indonesia, the switch was preceded by a national polio campaign that ensured as many infants as possible were vaccinated with existing stocks of tOPV before the switch and occurred concurrently with planning for the introduction of inactivated polio vaccine (IPV; IPV was introduced in late 2016). By comparison, the INSP was divided into the following stages: [1] developing management structures and committees (including developing working groups for planning, implementation, logistics, and monitoring and evaluating the switch); [2] budget (including securing funding sources for the national switch); [3] supply (including ensuring bOPV was available at all points of service and HFs and that tOPV was collected and disposed of after the switch); and [4] implementation (including tOPV disposal and disposal-site selection, training materials and preparation, and switch monitoring). To estimate the cost for the switch that could be applicable to similar settings, we mapped the activities within the 4 stages of the INSP to the 4 phases of the WHO global switch guidelines (Table 2); however, as there is not a one-to-one mapping between the activities in the WHO global switch guidelines and those in the INSP, we report the costs by the 4 WHO switch phases rather than by individual activity. The cost of purchasing bOPV was not included in the estimate because this was considered to be a cost transfer from the purchase of tOPV (ie, purchased bOPV was replacing tOPV that would have been purchased in the absence of the switch).

**Table 2.** Comparison of World Health Organization Global Switch Guidelines and Indonesia Switch National Plan by Phase

Phase	WHO global switch guideline activities [6]	Indonesian switch national plan activities [5] (activity no. in parentheses)
Phase I: Plan (July 2015–December 2015)	1. Meetings to select a national switch day; form subcommittees on vaccine supply, communications, logistics, process monitoring, and reporting; identify points of contact; and establish an operations center to coordinate activities on all levels (national, regional, district and facility)	Management committees/structure: Development of 5 working groups: working group on planning, working group on implementation, working group on logistics, working group on communication, and working group on monitoring and evaluation (1.0)
	2. Meetings to establish the national switch validation committee	National Switch Validation Committee: establishment of a committee to validate the switch (1.2.1)
	3. Conduct an analysis on the supply and distribution of OPV, licensing vaccine, establishing private sector provision of OPV, establishing communications of vaccine, establishing disposal of waste, establishing whether existing expertise exists	Supply assessments; bOPV procurement and distribution plan (3.1.4)
	4. Draft the national switch plan	Workplan and timeline of the switch (1.1.3)
Phase II: Prepare (August 2015–March 2016)	1. Secure all funds to hire additional staff and to manage logistics, assess tOPV inventory, costs for waste management, and training.	Budget: funding and resources (2.0)
	2. Develop communication with stakeholders, cold chain personnel, logisticians, and health workers.	Implementation preparation: communication (4.3)
	3. Develop all training material	Implementation preparation: training Materials and preparation (4.4)
	4. Includes travel to assess cold-chain capacity or any communications involving assessment of cold-chain capacity.	Implementation preparation: logistics, cold-chain capacity (4.1.1)
	5. Develop strategy or guidelines for disposing of tOPV. Select the official disposal sites.	Implementation preparation: tOPV disposal policy and monitoring and disposal site selection (4.1.2)
Phase III: Implement Switch (March 2016–May 2016)	1. Develop roles and responsibilities of switch monitors.	Subnational switch committees (1.1.1.1)
	2. Distribution of bOPV at designated time (suggested at 2 weeks) before switch.	Supply: bOPV procurement and distribution plan/private sector (3.1.4, 3.1.5)
	3. Provide a full day of training on the switch.	Implementation preparation: training materials and preparation (4.4)
	4. Remove all tOPV from cold chain and disposal. Use a sticker to identify any tOPV for disposal.	Implementation preparation: switch monitoring, monitoring process (4.3.5)
Phase IV: Validate (April 2016–June 2016)	1. Identify sites to be validated that tOPV has been removed.	tOPV disposal policy and monitoring and disposal site selection (4.1)
	2. Record all tOPV information.	tOPV disposal policy and monitoring and disposal site selection (4.1)
	3. Dispose of tOPV that remains through use of contingency plan.	tOPV disposal policy and monitoring and disposal site selection (4.1)
	4. Compile report of disposal to validation committee.	Implementation preparation: switch monitoring (4.3.5)

Abbreviations: bOPV, bivalent oral polio vaccine; OPV, oral polio vaccine; tOPV, trivalent oral polio vaccine.

### Role of National Expanded Program on Immunization, Provinces, Districts, and Cities in Switch Implementation

The National EPI office was responsible for coordinating all switch activities throughout the time period of the switch phases from July 2015 to June 2016. The responsibilities during the switch planning phase included coordination meetings and assistance with identifying leaders for the switch throughout Indonesia’s 34 provinces. Provincial health offices served as liaisons between Indonesia’s vaccine manufacturer and district and city health offices, ensuring that all vaccines (including tOPV) were adequately stocked until the implementation of the switch and that sufficient bOPV was available for districts and cities. In turn, district and city offices provided an adequate supply OPV to HFs [13]. All levels did not participate equally in all phases of the switch. In particular, participation in the validation phase varied across the sampled provinces. In Bali, provincial and district/city health offices participated in validation-phase activities, whereas only provincial health offices participated in the validation phase in West Sumatra and NTT provinces.

### Data Collection Procedures

Structured questionnaires were developed to collect data on the resource input types used to execute each phase of the switch, including personnel, supplies, equipment, training, travel (including vehicle use), and contracted services. The questionnaires were piloted at the provincial and district health offices in Jakarta and within a public clinic and revised based on the pilot results. The revised questionnaires were administered at the national EPI office, 3 provincial health offices, 3 district health offices, 3 city health offices, and 12 HFs. Respondents included personnel in the selected health facilities (n = 12), district or city health offices (n = 6), provincial health offices (n = 3), national EPI office (n = 1), and national Biofarma office (n = 1; n = 23 respondents in total). The questionnaire was administered to the same respondent for each phase of the switch. Data were collected on the quantity and price of resources reportedly used for each input category. We only examined incremental resources expended for the switch that were in addition to the resources reportedly used for regular immunization program activities. Questionnaires were administered in Bahasa Indonesia by a team of data collectors from the Ministry of Health’s Policy Unit with technical assistance from US Centers for Disease Control and Prevention staff during June–August 2016. Data were translated into English and entered and analyzed in Excel.

### Cost Analysis

The cost analysis was conducted from the perspective of the Indonesian government, including all sources of funding. We measured incremental economic costs, including all resources reportedly used for the 4 WHO switch phases, including financial outlays and use of existing or in-kind resources. The time frame and analytic horizon for the cost analysis were defined as the duration of the national switch in Indonesia, including the reported timeline for all 4 phases (July 2015 to June 2016). We calculated the costs for each sampled unit (national, province, city/district, and HF), switch phase, and resource input type. Vehicle and equipment costs were annuitized using a discount rate of 3%. All costs are presented in nominal US dollars (USD) using the average exchange rate with Indonesian rupiah (IDR) for the months of each switch-phase time period (planning phase: 13824 IDR to 1 USD; preparation phase: 13762 IDR to 1 USD; implementation phase: 13255 IDR to 1 USD; validation phase: 13311 IDR to 1 USD) [18]. Personnel costs were considered to be those costs associated with personnel working directly with the switch. This includes time spent in meetings and executing switch activities as defined in Table 2. Personnel costs were calculated as the percentage of time an individual reported working on a particular activity multiplied by the monthly wages and benefits that person received. Volunteer time for activities was valued by applying a monthly salary for a commensurate government personnel level to the volunteer’s time based on the responsibilities of that person. Supplies and materials donated to the intervention were valued at reported market prices.

### Subanalysis: Hypothetical Scenarios for Aggregate Switch Costs by Sampled Province and for Indonesia’s 34 Provinces

Although our convenience sample of provinces, cities/districts, and HFs does not permit statistically valid extrapolation of the cost-analysis results, as a hypothetical scenario analysis we explored what the aggregate switch costs for Indonesia might have been if the remaining 31 provinces had costs similar to those observed in our sample. We first estimated the total switch costs in each of our 3 sampled provinces for 3 scenarios based on applying the median, minimum, and maximum HF cost in each province to all HFs in that province, then applying the city health office costs to all cities in the province, the district health office costs to all districts in that province, and finally adding the reported provincial health office costs. Based on these median, minimum, and maximum scenarios for each of the 3 provinces, we then multiplied each scenario cost by the 31 remaining provinces and added the reported national-level costs for the EPI program to each to obtain an estimate of aggregate switch costs for Indonesia as a whole. The purpose of this subanalysis was to provide a hypothetical range of costs against which to compare the national switch budget that was developed to advocate for and secure funds for the switch in Indonesia ([13], Annex 4).

Data were requested from Biofarma, a parastatal company, on their resource inputs for the switch; however, the data received were incomplete and were not used in our final analysis. For example, Biofarma respondents declined to provide information on salaries and the exact number of staff involved at each level of its operations in relation to the switch. These staff costs in some cases were based on service contracts between Biofarma and other companies for operations related to the switch at the district, city, and HF levels, which Biofarma considered to be procurement-sensitive information. Our analysis, therefore, does not include Biofarma’s resource contributions to the switch.

## RESULTS

### Costs by Geographic Location, Health System Level, Switch Phase, and Resource Input

The estimated national-level EPI program costs were $46 791, 45% of which was incurred during the switch-planning phase (Table 3). Estimated costs ranged from $9062 to $34 256 at the province level among the 3 provinces surveyed, from $4576 to $11 936 among the 3 district-level health offices surveyed, and from $3488 to $29 175 among the 3 city-level health offices surveyed. There was substantial variation in the reported costs by phase of the switch across sampled provinces, health-system levels, and HF types (Table 3). In our sample, there was no consistent relationship between costs borne by the provincial-level health office and those borne by city-level and district-level health offices; in West Sumatra, the provincial-level costs were higher than the city/district costs, whereas in Bali and NTT at least 1 of the sampled cities/districts had total switch costs exceeding those at the provincial level. Summed across all switch phases, personnel time represented the largest share of costs at each level in our sample, with the exception of the West Sumatra District Health Office for which travel costs (including per diem) was the largest resource input (**Supplementary Table 1**). No training costs were reported at any level.

**Table 3.** Estimated Switch Costs by Health System Level and Phase (Study Sample)

Health-system level	Phase 1 (Plan)	Phase 2 (Prepare)	Phase 3 (Implement)	Phase 4 (Validate)	Total Phases 1–4
National					
EPI program	$20958	$18514	$6077	$1242	$46791
Bali Province					
Provincial health office	$4081	$1510	$2951	$519	$9062
District health office (n = 1)	$5876	$5095	$757	$208	$11936
City health office (n = 1)	$1668	$1490	$294	$36	$3488
West Sumatra Province					
Provincial health office	$12112	$15756	$6060	$328	$34256
District health office (n = 1)	$1043	$2427	$1106	$0	$4576
City health office (n = 1)	$3390	$2754	$280	$0	$6424
NTT Province					
Provincial health office	$3071	$2405	$2964	$1132	$9572
District health office (n = 1)	$1204	$3886	$739	$0	$5829
City health office (n = 1)	$16454	$11063	$1657	$0	$29175

### Costs by Health Facility Characteristics

Median switch costs per HF varied by geography and ownership characteristics (Table 4). Median switch costs per HF were higher for rural ($669) than for urban HFs ($244), and higher for public ($594) than for private HFs ($119). Median switch costs per HF were highest in the medium-OPV3-coverage West Sumatra province ($704), followed by the low-OPV3-coverage NTT province ($513), with the lowest median HF cost in the high-OPV3-coverage Bali province ($31). Reported HF switch costs ranged from as little as $31 to as much as $2193 for the planning, preparation, and implementation phases combined. The HF with the highest reported switch cost reported substantially higher costs for the preparation and implementation phases ($649 for preparation and $897 for implementation) compared with other HFs (minimum $3 for preparation and $510 for implementation).

**Table 4.** Switch Costs per Health Facility by Geography and Ownership Characteristic (Study Sample)

**Facility characteristic**	**Median total switch cost**	**Minimum switch cost**	**Maximum switch cost**
Rural health facilities (n = 6)	$669	$31	$2193
Urban health facilities (n = 6)	$244	$90	$629
Public health facilities (n = 8)	$594	228	$2193
Private health facilities (n = 4)	$119	$31	$467
Bali health facilities (n = 4)	$204	$31	$777
West Sumatra health facilities (n = 4)	$704	$90	$2193
NTT health facilities (n = 4)	$513	$282	$629

Abbreviation: NTT, Nusa Tenggara Timur.

### Subanalysis: Hypothetical Scenarios for Aggregate Switch Costs by Sampled Province and for Indonesia’s 34 Provinces

Hypothetical scenarios for aggregate switch costs are presented based on the cost scenarios derived from the convenience sample of three provinces (Tables 5**and**6). This subanalysis is intended as context for the INSP proposed switch budget and should be interpreted with caution considering the small convenience sample of provinces, cities/districts, and HFs, which were not intended to be representative of all such units in Indonesia. These hypothetical scenarios produced estimated total per-province costs ranging from $113 567 (Bali, minimum scenario) to $857 911 (West Sumatra, maximum scenario), with a median per-province cost across scenarios of $278 367 (NTT, minimum scenario). For the estimated total province cost, the minimum scenario refers to the extrapolation of the lowest cost HF for all HF costs in the province, whereas the maximum scenario refers to the extrapolation of the highest cost HF for all HF costs in the province. In each scenario, the sampled city and district costs were applied to all cities and districts in the province. It is noteworthy that the INSP proposed switch budget of $16.9 million falls within the range of the hypothetical scenarios of aggregate estimated costs for Indonesia as a whole, which ranged from $4 076 446 (Bali, minimum scenario) to $28 120 700 (West Sumatera, maximum scenario), with a median aggregate cost across scenarios of $9 185 270 (NTT, minimum scenario).

**Table 5.** Estimated Aggregate Switch Costs for Each Sampled Province

Province	Median scenario (assuming median health facility cost in each province)	Minimum Scenario (assuming minimum health facility cost in each province)	Maximum Scenario (assuming maximum health facility cost in each province)
Bali	$143987	$113567	$244848
West Sumatra	$366565	$163947	$857911
NTT	$374695	$278367	$422694

Estimates are hypothetical and intended to provide context for comparison with Indonesia’s switch budget.

**Table 6.** Estimated Aggregate Switch Costs for Indonesia (34 Provinces + National Expanded Program on Immunization Costs)

Province used as basis for extrapolation to other 31 provinces	Median scenario (assuming median health facility cost in each province)	Minimum scenario (assuming minimum health facility cost in each province)	Maximum scenario (assuming maximum health facility cost in each province)
Bali	$5348842	$4076446	$9115744
West Sumatra	$12248758	$5638246	$28120700
Nusa Tenggara Timur	$12500792	$9185270	$14628964

^a^Estimates are hypothetical and intended to provide context for comparison with Indonesia’s switch budget.

## DISCUSSION

Contrary to the conventional wisdom that vaccine withdrawal or recall does not entail additional costs, global partners thought the tOPV–bOPV switch would require additional resources, as evidenced by the global switch guidelines that include recommendations for resource mobilization as well as the financial assistance provided by GPEI to country governments to support the hypothesized extra costs of switch implementation [5]. Indonesia was one of the largest recipients of the GPEI switch funds, and its own national switch budget was >$16 million, including both domestic and external funding. Although global partners and country governments hypothesized that the switch would require additional resources, our study is the first to examine whether there is evidence to support this hypothesis. The findings of this case study provide evidence of the additional cost at the country level of a global synchronized vaccine withdrawal event, which to our knowledge does not exist in the published literature on vaccine costs. Although our case study examined only a convenience sample of provinces, cities, districts, and HFs in Indonesia, our results represent the first country-level data on the reported costs of this globally synchronized vaccine switch.

Our findings suggest that the majority of the switch costs were borne at the subnational levels. In part, this is expected, given the decentralized nature of Indonesia’s government and health system; however, it highlights the important contributions (many in-kind) by provincial, city, and district governments and by HF staff, as well as the importance of adequate budgeting for subnational coordination and implementation for future switches. In our study sample, the majority of switch costs were incurred during the planning and preparation phases, primarily reflecting personnel time spent on planning and coordination meetings. Costs during the implementation and validation phases were lower than might have been expected for the core activities of implementing and validating the switch. Validation-phase involvement was limited to some health-system levels (eg, there were no validation-phase costs reported at the HF level), which may also explain the smaller share of costs for activities in this phase.

### Lessons Learned and Implications for Policy and Practice

Our findings suggest that in-kind contributions of countries to program costs of the switch were substantial, even though these are not traditionally reflected in financial costs presented in budgets. The variability in estimated switch costs across provinces, districts, and cities suggests a need for flexibility in budgeting for immunization activities involving multiple health-system levels. Attempting to force a rigid approach to budgeting upon all levels (eg, a flat amount per administration unit) would likely lead to suboptimal performance, particularly in a country as varied as Indonesia in which different levels of government may play different roles in planning, implementation, and monitoring across different areas.

Although training was a major activity outlined in the global guidelines and Indonesian switch plan and budget, our respondents reported no training costs incurred at any level (**Supplementary Table 1**). This may reflect prevailing perceptions around training as formal classroom delivery of new content at a dedicated time and place, for which transport costs and a per diem are paid to participants. Information about the switch that may have been conveyed through in-service training methods, such as at monthly meetings of HF staff at district or city health offices or during supervision visits, might not have been perceived as training by respondents. Training on the switch might also have been incorporated into trainings on other topics (eg, IPV introduction and planning for a national immunization day) such that respondents did not report any training on the switch per se. The reported limited investment in training may also reflect resource constraints because the external resources that Indonesia received from GPEI were less than requested. One implication for future vaccine switches and withdrawals may be that when resources are constrained, countries can find more efficient options than formal training (with its associated costs for transport, per diem, and venue) for transmitting information to subnational health authorities and HF staff.

We did not find a clear correlation between costs and vaccination coverage; although previous studies of immunization service delivery costs have used immunization program performance as a basis for sampling, our results suggest that coverage as a measure of immunization program performance may not be an informative basis for resource allocation at the subnational level to support similar vaccine switches or withdrawals in the future. Although some costs (eg, cold chain) are shared, the costs of the switch appear to have not been associated with vaccination coverage, perhaps reflecting the difference in core activities (eg, planning meetings vs vaccine administration).

### Limitations

Our findings should be interpreted in light of several limitations. We used convenience sampling for provinces, districts/cities, and HFs; therefore, the site sample may not be representative of all such sites in Indonesia. We did not collect data on the population served for the HFs in our sample, and thus we could not compare costs across HFs based on catchment population. Also, we did not include either the cost of bOPV distribution to all peripheral levels or Biofarma’s costs of destroying tOPV remaining after the switch due to missing information and quality of data received. Furthermore, our results may be subject to recall bias, as is possible with all retrospective cost data collection relying on respondents to recall time spent or resources used, possibly leading to personnel costs or other expenses being overestimated or underestimated. Respondents’ answers were solicited by self-report and not validated through document review (eg, accounting, stock, or health records) because the cost analysis was designed to be a program evaluation and not an audit. Moreover, respondents were unable to provide cost information for some activities when such information was deemed to be procurement-sensitive (eg, cost of Biofarma distribution service contracts in each province); total costs are therefore underestimated. In addition, the switch in Indonesia was implemented simultaneously with several other immunization interventions, including IPV introduction and national immunization week 2016. It is therefore possible that some incremental costs of the switch are underreported by respondents because they were associated with or attributed to these parallel activities. Our analysis adopted a government perspective and therefore does not include contributions made by external partners that were not channeled through the government (eg, GPEI funding to support WHO national program officers).

Furthermore, we were unable to provide insight into potential shared costs with IPV introduction. Although IPV was scheduled to be introduced in Indonesia in June 2016, its introduction was delayed due to delivery constraints and did not overlap with the implementation of the tOPV-to-bOPV switch (which covers the period from July 2015 to June 2016); however, some preparatory activities for IPV introduction, such as training or planning meetings, may have overlapped with the switch. Although our questionnaire and data-collection team specifically asked about additional resources used only for the switch, it is possible that respondents may have attributed overlapping activities fully to IPV introduction or fully to the switch, leading to either an underestimate or overestimate of switch costs.

## CONCLUSIONS

This cost analysis of the tOPV-to-bOPV switch in Indonesia provides evidence of the reported resource contributions at all levels of the Indonesian health system to implement the switch. For all phases of the WHO global switch guidelines, we documented resources reportedly used for the phases of the switch, providing another lens on programmatic reports of the extent of switch planning and implementation. The initial INSP budget estimates for the switch were in the range of aggregate switch costs we present here based on a convenience sample of provinces, cities/districts, and HFs, although our cost estimates may be underreported due to recall bias and the integration of the switch with other concurrent vaccination activities. The empirical retrospective economic cost estimates presented here indicate that, although there was likely considerable variation in the resources used as reported to carry out the switch across provinces, cities, districts, and HFs, overall the switch’s resource needs were likely manageable. This suggests that the resource requirements for the future withdrawal of bOPV are likely to be similarly manageable at a country level.

## Supplementary Data

Supplementary materials are available at *The Journal of Infectious Diseases* online. Consisting of data provided by the authors to benefit the reader, the posted materials are not copyedited and are the sole responsibility of the authors, so questions or comments should be addressed to the corresponding author.

## Supplementary Material

Supplementary_Table1

## Notes

***Acknowledgments.*** The authors thank Rihlah Romdinah and Yudi Helfi for their assistance with data collection and translation.

***Disclaimer.*** The findings represent the personal views of the authors and not the official position of the US Centers for Disease Control and Prevention or the Indonesian Ministry of Health.

***Financial support.*** This work was supported by the US Centers for Disease Control and Prevention.

***Supplement sponsorship.*** This work is part of a supplement coordinated by the Task Force for Global Health with funding provided by The Bill and Melinda Gates Foundation and the Centers for Disease Control and Prevention.

***Potential conflicts of interest.*** All authors: No reported conflicts. All authors have submitted the ICMJE Form for Disclosure of Potential Conflicts of Interest. Conflicts that the editors consider relevant to the content of the manuscript have been disclosed.
