# Supplementary material for: Resource Needs for the Trivalent Oral Polio to Bivalent Oral Polio Vaccine Switch in Indonesia
Source: J Infect Dis. 2017 Jun 30;216(Suppl 1):S209–16. doi: 10.1093/infdis/jix073 (PMC5853418; doi:10.1093/infdis/jix073)
Supplement: Supplementary_Table1 [file jix073_suppl_supplementary_table1.docx]

**Supplemental Table 1. Switch costs by resource input***

| **Health System Level** | **Personnel** | **Materials & Supplies** | **Equipment** | **Travel (incl. per diem)** | **Vehicles** | **Training** |
| --- | --- | --- | --- | --- | --- | --- |
| **National** |  |  |  |  |  |  |
| EPI Program | $40,036 | $3,735 | $373 | $2,647 | $0 | $0 |
| **Bali Province** |  |  |  |  |  |  |
| Provincial Health Office | $4,173 | $491 | $45 | $4,003 | $350 | $0 |
| District Health Office (n=1) | $11,445 | $298 | $166 | $16 | $10 | $0 |
| City Health Office (n=1) | $3,018 | $245 | $135 | $30 | $59 | $0 |
| Health facilities (n=4) | $782 | $161 | $14 | $24 | $236 | $0 |
| **West Sumatra Province** |  |  |  |  |  |  |
| Provincial Health Office | $24,569 | $3,900 | $145 | $55 | $5,587 | $0 |
| District Health Office (n=1) | $1,122 | $942 | $7 | $2,421 | $85 | $0 |
| City Health Office (n=1) | $5,997 | $214 | $164 | $20 | $29 | $0 |
| Health facilities (n=4) | $1,080 | $1,296 | $67 | $924 | $325 | $0 |
| **NTT Province** |  |  |  |  |  |  |
| Provincial Health Office | $3,550 | $502 | $2,067 | $3,072 | $381 | $0 |
| District Health Office (n=1) | $5,398 | $230 | $188 | $11 | $1 | $0 |
| City Health Office (n=1) | $28,883 | $93 | $180 | $11 | $8 | $0 |
| Health facilities (n=4) | $1,770 | $100 | $31 | $20 | $16 | $0 |
